# Dietary mercury intake, the *IL23R* rs10889677 polymorphism, and the risk of gastric cancer in a Korean population: a hospital-based case-control study

**DOI:** 10.4178/epih.e2024051

**Published:** 2024-05-21

**Authors:** Ji Hyun Kim, Madhawa Gunathilake, Jeonghee Lee, Il Ju Choi, Young-Il Kim, Jeongseon Kim

**Affiliations:** 1 National Cancer Center Graduate School of Cancer Science and Policy, National Cancer Center, Goyang, Korea; 2 Center for Gastric Cancer, National Cancer Center Hospital, National Cancer Center, Goyang, Korea

**Keywords:** Stomach neoplasms, Diet, Mercury, Interleukin-23 receptor, Polymorphism, Susceptibility

## Abstract

**OBJECTIVES:**

Mercury can stimulate immune responses through T helper 17 (Th17). The gene *IL23R* is a key factor in Th17 function, which may also contribute to digestive tract diseases. The aim of this study was to identify the associations between dietary mercury and gastric cancer (GC) and to investigate whether the *IL23R* rs10889677 polymorphism modifies those associations.

**METHODS:**

This case-control study included 377 patients with GC and 756 healthy controls. Dietary mercury intake (total mercury and methylmercury) was assessed using a dietary heavy metal database incorporated into the food frequency questionnaire. *IL23R* genetic polymorphism rs10889677 (A>C) was genotyped. Odds ratios (ORs) and 95% confidence intervals (CIs) were calculated using unconditional logistic regression models with adjustments for potential confounders.

**RESULTS:**

A higher dietary methylmercury intake was associated with an elevated risk of GC (OR for the highest vs. lowest tertile [T_3_ vs. T_1_], 2.02; 95% CI, 1.41 to 2.91; p for trend <0.001). The *IL23R* rs10889677 reduced the risk of GC in individuals who carried at least 1 minor allele (OR, 0.62; 95% CI, 0.46 to 0.83; p=0.001; AC/CC vs. AA). Individuals with a C allele exhibited a lower susceptibility to GC through methylmercury intake than those with the AA genotype (OR for the T_3_ of methylmercury and AA carriers, 2.93; 95% CI, 1.77 to 4.87; and OR for the T_3_ of methylmercury and AC/CC genotype, 1.30; 95% CI, 0.76 to 2.21; p-interaction=0.013).

**CONCLUSIONS:**

Our findings suggest that a genetic polymorphism, rs10889677 in *IL23R*, plays a role in modifying the association between dietary methylmercury intake and the risk of GC.

## GRAPHICAL ABSTRACT

[Fig f2-epih-46-e2024051]

## Key Message

This study aimed to investigate the associations between dietary mercury and gastric cancer (GC) and to determine whether the *IL23R* rs10889677 polymorphism, located within a predicted binding site for microRNA-lethal-7, may modify these associations. A higher dietary methylmercury intake was associated with an increased risk of GC, while the *IL23R* rs10889677 polymorphism may modify the detrimental effect of dietary methylmercury on gastric carcinogenesis.

## INTRODUCTION

Gastric cancer (GC) was one of the most diagnosed cancers and a major cause of cancer-related deaths worldwide in 2020, ranking fourth in incidence and fifth in mortality [1]. Despite a continuous declining trend in GC incidence and improved survival rates, the burden remains high in East Asian countries, particularly in Korea [1,2]. Therefore, it is crucial to conduct further epidemiological studies to better understand the factors underlying gastric carcinogenesis, including environmental and genetic factors.

As an environmental contaminant, mercury is a persistent and toxic heavy metal harmful to health. It can originate from various emission sources, transfer through different environmental mediums, undergo complex biogeochemical cycling, and accumulate in animals and plants [3]. Mercury-induced toxicity is not confined to a single cellular target; it can cause extensive damage throughout the body, including neurotoxicity, nephrotoxicity, and gastrointestinal toxicity, resulting in ulceration and hemorrhage [4-6]. Diet is the predominant route of mercury exposure in humans, with fish being the main source of mercury intake [7-9]. Scientific literature suggests that regular exposure to mercury or its most toxic organic form, methylmercury (derived from methylation of inorganic mercury by microorganisms), may adversely contribute to digestive disorders including chronic gastritis and colorectal cancer [5,10-12].

Oxidative stress is a common pathway through which genotoxicity is induced [13]. The reactive oxygen species triggered by mercury can cause lesions of the gastric mucosa, damage DNA, and subsequently disrupt gene regulation, cell signal transduction, and cell growth, which can ultimately lead to gastric carcinogenesis and metastasis [14]. The induced formation of free radicals has been implicated in promoting inflammation and damage in the context of autoimmunity [15]. In addition, xenobiotics can stimulate immune responses by binding to immune cell receptors, which are expressed by many immune cells (e.g., T helper 17 [Th17] subsets) [16-18]. While the exact etiology remains elusive, heavy metals (a prominent category of xenobiotics) have been implicated in the alteration of immune cell responses in exposed individuals, thereby contributing to disease susceptibility (e.g., autoimmune diseases and cancers), particularly when accompanied by inflammation-induced sensitization [18,19].

Interleukin 23 receptor (IL-23R) is a key player in the pro-inflammatory signal transduction pathway of the IL-23/IL-17 axis (IL-23→IL-23R→STAT_3_→Th17→IL-17/IL-17F) and is recognized for its crucial role in inflammatory diseases and cancers [20,21]. It has been hypothesized that genetic variants within this pathway modify cancer risk. Several functional variants in the *IL23R* gene have been reported to impact cancer susceptibility [22]. Notably, rs10889677 is situated in the 3´-untranslated region (3´-UTR) of *IL23R* and is located within a predicted binding site for microRNA lethal-7 (miR-let-7) [23]. It has been reported that nucleobase substitution (A> C) may increase the binding affinity of miR-let-7 and can further affect posttranscriptional regulation of *IL23R* [23]. Several types of cancers (e.g., stomach, colorectum, ovary, breast, lung, nasopharynx, and bladder) have been investigated in relation to this genetic polymorphism, and pooled estimates from meta-analyses noted a reduced risk of cancer among AC/CC genotypes when compared to AA genotypes [22-24]. However, the findings regarding this variant in digestive tract cancers remain unclear [20,23,25-29], and previous studies did not include the potential effects of dietary factors when analyzing this variant [27].

To our knowledge, there is a paucity of epidemiologic evidence examining the impact of dietary mercury exposure or *IL23R* polymorphism on cancer risk. Moreover, the combined effect of dietary mercury intake and the *IL23* variant on gastric carcinogenesis has not been investigated thus far. Therefore, this study investigated the association between dietary mercury intake or *IL23R* rs10889677 polymorphism and GC risk, and explored whether there was an interaction between dietary mercury intake and the *IL23R* variant in relation to GC.

## MATERIALS AND METHODS

### Study participants

**Figure 1.**
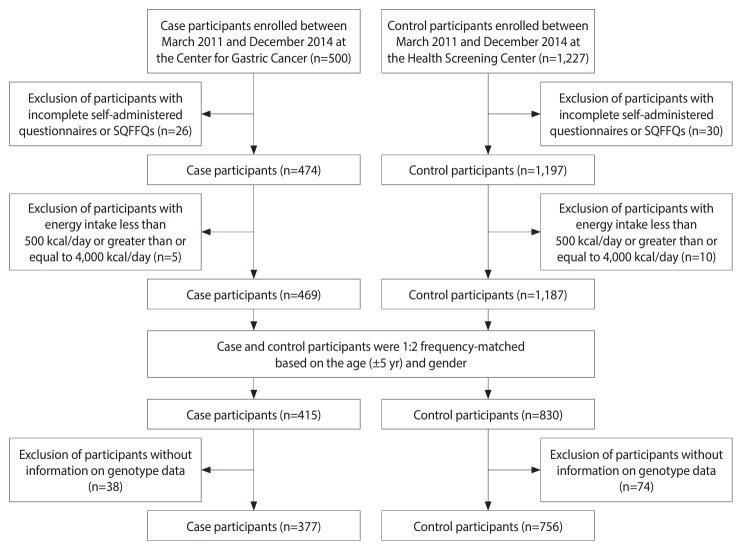
Flowchart of the participant selection process. SQFFQs, semi-quantitative food frequency questionnaires.

To conduct a case-control study, we recruited participants from the National Cancer Center Hospital (NCC) in Korea between March 2011 and December 2014. Case participants included those diagnosed with GC within 3 months prior to enrollment and confirmed as invasive carcinoma in the NCC’s Center for Gastric Cancer. Patients with severe chronic diseases and pregnant or breastfeeding women were excluded. Participants who underwent health-screening examinations at the NCC’s Cancer Prevention and Detection Center, which confirmed that they did not have a history of cancer or other severe comorbidities, were recruited as controls. We used age and gender to match controls and cases at a ratio of 2:1. A total of 756 controls and 377 cases with available genotype data were included for analysis (Figure 1). Detailed descriptions of participant recruitment have been outlined elsewhere [30].

### Data collection

Information on socio-demographic characteristics and lifestyle factors was collected from each participant using a self-administered questionnaire. The status of *Helicobacter pylori* infection was determined histologically or serologically with at least a positive result on a rapid urease test (Pronto Dry, Medical Instruments Corp., Solothurn, Switzerland). The participants’ dietary intake data over the 12 months prior to their interview was obtained by a well-trained dietary interviewer using a validated 106-item semiquantitative food frequency questionnaire [31]. The determination of daily total energy and detailed food item intake were based on a combination of intake frequency (never or rarely, 1 times/mo, 2-3 times/mo, 1-2 times/wk, 3-4 times/wk, 5-6 times/wk, 1 times/day, 2 times/day, and 3 times/day) and portion size (small, medium, and large), using a Computer Aided Nutritional analysis program (CAN-PRO 4.0, Korean Nutrition Society, Seoul, Korea). The daily food consumption information was subsequently linked to a database of dietary heavy metals, encompassing total mercury in all forms, including methylmercury. The dietary heavy metal database utilized in this study was developed by analyzing heavy metal content in samples of foods predominantly consumed by the Korean population. The samples were collected from markets in Korea and analyzed using methods specified in the Korean Food Standard Codex. The gold amalgamation method was used to detect total mercury, and gas chromatography with an electron capture detector was used to detect methylmercury. The participants’ food consumption data (g/day) were linked to the mercury levels (μg/g) of listed food items to estimate daily mercury consumption (μg/day). A detailed explanation of the database has been provided elsewhere [32,33].

### Genotyping and single nucleotide polymorphism selection

Genomic DNA samples were extracted from the peripheral blood leukocytes of all participants. The genotyping was performed using an Affymetrix Axiom^®^ Exome 319 Array containing 318,983 variants (Affymetrix Inc., Santa Clara, CA, USA). Genotype imputation was performed using the Asian population (n=504) in the 1000 Genome haplotypes phase III integrated variant set release GRch37/hg19 (https://www.1000genomes.org/) as a reference panel. We used SHAPEIT (v2.r837) for phasing and IMPUTE2 (2.3.2) for single nucleotide polymorphism (SNP) imputation. After filtering for an imputation quality score (or INFO score) over 0.6, quality control criteria were applied. Detailed information on the genotyping and quality control steps is mentioned elsewhere [34,35].

The following quality control criteria were used for further exclusions: (1) SNPs with a low call rate (<97%), (2) individuals with a low genotype call rate (<97%), (3) SNPs with a minor allele frequency (MAF) <5%, (4) SNPs showing deviation from Hardy-Weinberg equilibrium (p-values <1 × 10^-6^), and (5) individuals closely related based on a pairwise identity-by-descent proportion (pi-hat >0.25). The current study focused on rs10889677 polymorphism, which is located in the 3´-UTR of *IL23R* and within a binding site for miR-let-7 [23].

### Statistical analysis

We assessed the differences between cases and controls in sociodemographic, anthropometric, and lifestyle factors and in total energy intake using the chi-square test for categorical variables and the Student t-test for continuous variables. Mercury intake was adjusted for energy using the residual method [36]. We classified dietary mercury intake into tertiles based on the distribution among controls. For the genetic association, we employed the codominant, dominant, and allelic inheritance models. To analyze associations among dietary mercury consumption, *IL23R* rs10889677, and the risk of GC, we constructed unconditional logistic models to estimate the odds ratios (ORs) and 95% confidence intervals (CIs), considering potential confounding factors. The covariates included continuous age and the categorical gender, body mass index (< 23, 23-< 25, or ≥ 25 kg/m^2^), smoking status (current-, ex-, or non-smoker), drinking status (current-, ex-, or non-drinker), physical activity (yes or no), education attainment (less than college or college and higher), income (< 200, 200-< 400, or ≥ 400 [×10,000 Korean won/mo]), and first-degree family history of GC (yes or no). Because the expression of IL-23 is closely associated with *H. pylori* infection [37,38], we included it in the final statistical model. Moreover, we investigated the dose-response effects of dietary mercury in relation to GC risk by using the median value within each tertile of dietary mercury intake to identify a test for trend. To test for the effect of the interaction between dietary mercury and the *IL23R* variant on GC risk, we employed a likelihood ratio test by comparing models with and without the interaction term (tertile categories mercury *SNP). All statistical analyses were conducted using SAS version 9.4 (SAS Institute Inc., Cary, NC, USA), and a 2-sided p-value < 0.05 indicated statistical significance.

### Ethics statement

This study was approved by the Institutional Review Board of the National Cancer Center Korea (No. NCC 2021-0181), and written informed consent was obtained from all participants prior to the examination.

## RESULTS

The general characteristics of the study participants and their dietary mercury intake are presented in Table 1. Compared to healthy participants, patients with GC exhibited a greater prevalence of positive *H. pylori* infection (92.6 vs. 61.4%), a first-degree family history of GC (20.4 vs. 12.6%), current smoking status (30.8 vs. 20.4%), and education less than college (75.8 vs. 44.2%). When compared to the controls, GC cases showed less adherence to regular physical activity (36.1 vs. 56.1%) and fewer had a monthly income ≥ 4,000,000 Korean won (23.3 vs. 32.7%). The case participants exhibited a higher daily caloric intake (1,925.2±611.9 vs. 1,717.3±546.9 kcal/day) and a greater mean dietary mercury intake (energy-adjusted total mercury: 14.0±2.3 vs. 13.7±2.4 µg/day; and methylmercury: 13.5±3.5 vs. 12.4±3.3 µg/day) than the controls.

The association between dietary mercury intake (total mercury and methylmercury) and GC risk is reported in Table 2. When compared to the lowest tertile (T_1_) of dietary total mercury, men participants in the highest intake tertile group (T_3_) were associated with an elevated risk of GC (model III: OR, 1.73; 95% CI, 1.11 to 2.68; p for trend=0.015). However, in participants overall, the increased OR due to dietary total mercury exposure observed in the crude model disappeared after adjusting for potential covariates. Among women, no clear concentration-response effects were observed with increasing tertiles of dietary total mercury. The results indicated an elevated risk of GC in the second tertile (T_2_) in both crude and fully adjusted models, but not in the T_3_. In terms of dietary methylmercury intake, an increased risk of GC was observed in participants overall (OR, 2.02; 95% CI, 1.41 to 2.91; p for trend < 0.001). In the gender-stratified analysis, the proportional association trends of methylmercury intake with GC risk remained consistent in both men (OR, 1.77; 95% CI, 1.14 to 2.74; p for trend=0.010) and women (OR, 2.80; 95% CI, 1.40 to 5.62; p for trend=0.001).

The association between *IL23R* polymorphism and the risk of GC is shown in Table 3. In the dominant model, we observed a reduced GC risk in individuals carrying at least 1 minor allele C of *IL23R* rs10889677 polymorphism (OR, 0.62; 95% CI, 0.46 to 0.83; AC/CC vs. AA). When stratifying the participants by gender, the inverse associations remained statistically significant in both men (OR, 0.61; 95% CI, 0.42 to 0.88; AC/CC vs. AA) and women (OR, 0.60; 95% CI, 0.36 to 0.99). In the allelic model, a reduced risk of GC was also observed among participants overall and men specifically, in those carrying the C allele (OR, 0.74; 95% CI, 0.59 to 0.93 for overall; and OR, 0.71; 95% CI, 0.53 to 0.95 for men). In the codominant model, a decreased risk of GC was found in the AC genotype, but not in the CC genotype, when compared to the AA genotype. This trend persisted among overall participants as well as in both men and women (Supplementary Material 1).

We also investigated the associations between dietary mercury intake and the risk of GC using the dominant model of the *IL23R* rs10889677 variant, with the T_1_ of mercury intake and the homozygous AA genotype set as the reference. In the analysis of total mercury intake among overall participants, non-significant associations were identified for all groups based on tertiles of total mercury intake and the dominant model, as well as for the interaction between total mercury and the *IL23R* rs10889677 variant in relation to GC. This trend persisted in the gender-stratified analysis, except for a borderline significant reduction in the risk of GC noted among men with the lowest total mercury and AC/CC genotypes versus the AA genotype (Table 4). In the analysis of dietary methylmercury intake among total participants, individuals carrying the C allele showed a comparatively lower susceptibility to GC from methylmercury than those with the AA genotype (OR for the T_3_ of methylmercury and AA genotype, 2.93; 95% CI, 1.77 to 4.87; and OR for the T_3_ of methylmercury and AC/CC genotype, 1.30; 95% CI, 0.76 to 2.21; p-interaction=0.013). When stratified by gender, a higher intake of methylmercury and the presence of a C allele exhibited an attenuated risk of GC in both men and women. However, no significant interaction was found between dietary methylmercury and the *IL23R* rs10889677 variant for GC risk (Table 5).

## DISCUSSION

The present study investigated the association between dietary mercury intake and GC risk, considering the *IL23R* rs10889677 genetic variant among the Korean population. The key findings were (1) a higher dietary intake of methylmercury was significantly associated with an elevated risk of GC; (2) participants carrying the C allele for *IL23R* rs10889677 showed a protective effect on GC; and (3) the adverse impact of dietary methylmercury on GC tends to be less prominent among participants with the C allele for *IL23R* rs10887677.

We observed that a higher intake of mercury was associated with an elevated risk of GC. The effect of mercury on digestive tract diseases has been reported in several previous studies. A case-control study in Korea using the same dietary heavy metal database also demonstrated an increased risk of colorectal cancer among those who ingested greater amounts of dietary mercury [11]. In a study of 152 patients with chronic gastritis and 149 healthy controls in China, mercury levels in hair showed positive correlations with the severity of chronic gastritis among patients and with seafood intake among controls [10]. Another community-based cross-sectional study of 80 participants was conducted in the Canadian arctic region, where freshwater fish or seafood served as key food sources of methylmercury [5]. In this study, a model of gastric carcinogenesis incorporating intermediate endpoints (e.g., intestinal metaplasia, atrophy, and severe chronic gastritis) was utilized, and the ORs for severe chronic gastritis and atrophy reached their highest levels when hair-methylmercury levels exceeded 1 μg/g, particularly in conjunction with the lowest selenium intake [5]. Furthermore, mercury has been identified as a toxic compound that may promote cancer by inhibiting gap junction intercellular communications and producing inflammatory cytokines [39]. That evidence supports the hypothesis that mercury may contribute to inflammatory responses in the digestive tract, which can progress to carcinogenesis [5,10]. This also aligns with the findings of our study, which emphasized that a higher intake of dietary methylmercury was a significant risk factor for GC, likely due to the rapid absorption of highly lipophilic organic mercury in the gastrointestinal tract where a significant portion of the mercury accumulates in the human body [12,13]. A comprehensive review of epidemiological and experimental toxicology studies on cancer indicated that, although a plausible relationship between mercury-induced toxicity and cancer exists, there were inconsistencies across epidemiological studies (e.g., exposure assessment methods and cancer type). This highlights the need for further investigation [13].

Furthermore, the present study revealed a protective effect of the genetic polymorphism of the *IL23R* rs10889677 AC/CC or AC genotype on GC risk, but not for CC. According to the SNP database provided by the National Center for Biotechnology Information, a relationship between rs10889677 and GC risk has been documented in several studies, though the results were controversial. According to a case-control study comprising 1,010 cases of GC and 800 healthy controls, individuals carrying the AC or CC genotype were protected against GC when compared with those with the AA genotype (CC: OR, 0.47; 95% CI, 0.31 to 0.71 and AC: OR, 0.81; 95% CI, 0.66 to 0.99) [20]. In another study involving 500 cases and 500 controls, individuals with the CC genotype showed an elevated risk of GC (OR, 2.22; 95% CI, 1.27 to 3.87) compared to AA, whereas those with the AC or AC/CC genotype did not [25]. Non-significant associations with GC were observed in a study of 898 patients with GC and 992 controls (AC or CC) [26] and in another study with 479 cases of GC and 483 controls (AC/CC) [27]. Among case-control studies of other digestive tract cancers, the Chinese population with the AC or CC genotype exhibited a reduced risk of esophageal squamous cell carcinoma [40]. The AA genotype was identified as a risk factor for colorectal cancer in Iranian adults [23], while no correlation was observed in the Tunisian population [28]. Comprehensive findings from meta-analyses incorporating several types of solid cancers have demonstrated that the rs10889677 A> C may play a crucial role in the malignant transformation process; individuals with AC, CC, or AC/CC genotypes showed a lower risk of tumors than those with AA [22-24]. However, it should also be noted that the relationship of this genetic variant to prognosis in patients with GC may differ from its relationship to cancer incidence. One out of 2 studies following the mortality of patients with GC suggested an increased risk with each increment of the C allele (hazard ratio, 1.25; 95% CI, 1.05 to 1.49) [26], while another study reported that survival was not affected by their genotype [27]. A case-only study on breast cancer also showed that the C allele affected the age of cancer onset [41]. Therefore, prospective studies on both cancer incidence and prognosis are warranted to investigate how the *IL23R* genetic variant affects outcomes differently.

The possible biological mechanisms underlying the modification of cancer incidence by *IL23R* rs10889677 need to be explored. The *IL23R* gene encodes IL-23R, which plays a pivotal role in initiating, maintaining, and accelerating the IL-23/IL-17 inflammatory signal transduction pathway and is crucial for tumorigenesis [42-44]. Importantly, rs10889677 is a functional SNP located within the 3´-UTR of the *IL23R* and miRNA-mRNA hybridization site. It may alter the binding affinity of miR-let-7 and further downregulate *IL23R* gene expression, consequently inhibiting the translation of the IL-23R protein [23,24,45]. It has been shown that cancer-free adults with the rs10889677 AA genotype exhibited a higher expression of IL-23R in peripheral blood mononuclear cells relative to those with the AC/CC genotype. Indeed, the A allele carriers exhibited higher levels of regulatory T (Treg) cells *in vivo* and a lower T-cell proliferation rate *in vitro* when compared to C allele carriers [45]. It is also noteworthy that the imbalance between Th17 and Treg cells, 2 distinct CD4^+^ T cell subsets, can promote tissue inflammation by secreting the pro-inflammatory cytokine IL-17 and may ultimately contribute to carcinogenesis [46,47].

These findings provided a notable insight into the role of *IL23R* rs10889677 A>C in altering susceptibility to GC. This can likely be attributed to the reduction in IL-23R expression levels and the modulation of the IL-23/IL-17 inflammatory axis, both known to be implicated in the development of GC [20]. In addition, exposure to toxicants such as methylmercury may trigger immune dysfunction (e.g., cellular signals linked to the development of autoimmunity) and DNA damage, potentially increasing susceptibility to GC [14,18,19]. Collectively, we noted a significant trend wherein individuals carrying the C allele exhibited a comparatively lower susceptibility to GC from methylmercury intake than those with the AA genotype. Although the exact biological role remains unclear, our findings suggest that the *IL23R* rs10889677 polymorphism may interact with dietary methylmercury to stimulate pro-inflammatory cytokine responses, potentially contributing to the development of GC. Therefore, it is noteworthy that both low methylmercury intake and a specific polymorphism may play a protective role against GC.

The current study is one of the few studies investigating the effect of dietary mercury or a genetic polymorphism related to the IL17/IL23 inflammation pathway axis on the risk of GC. It also represents a novel attempt to explore the impact of an interactive effect between dietary mercury intake and the *IL23R* genetic variant on GC. Nevertheless, several limitations should be addressed. First, bias may be present due to the case-control study design. In terms of selection bias, the control participants were recruited from individuals who visited the clinic for a health check-up. They may have been more health-conscious and may have adopted healthier behaviors compared to patients with GC or those who were unwilling to receive health screening. Furthermore, our findings can be influenced by recall bias. The case participants may have recalled their past behaviors with greater accuracy since those factors were believed to be related to their disease. Accordingly, differences in the observed rates of exposure to risk factors between the cases and controls could be overstated. Nevertheless, the current study utilized a validated dietary questionnaire that was not influenced by prior knowledge of the research hypotheses [30]. Second, although the dietary mercury intake from each participant was meticulously linked with our heavy metal database, we could not validate the dietary mercury intake with mercury levels from biospecimens [48]. In future studies concerning GC risk, it is essential to validate the correlation between biospecimen mercury levels and dietary mercury intake. Lastly, while we suggested the genetic involvement of the IL17/IL23 pathway in the link between dietary mercury and GC, exploring a complex combination of genes in future studies is required for a thorough understanding of other genetic aspects in these biological processes [49,50].

In conclusion, we observed that a higher dietary intake of methylmercury was associated with an increased risk of GC, while *IL23R* rs10889677 polymorphism at the predicted miR-let-7 binding site exhibited a protective effect against GC. This genetic variant may modify the detrimental effect of dietary methylmercury on GC. Future large-scale prospective studies, incorporating biospecimen mercury levels and a wide array of genes related to miR-let-7 among diverse ethnicities, are warranted to unravel their roles in GC risk.

## Figures and Tables

**Figure f2-epih-46-e2024051:**
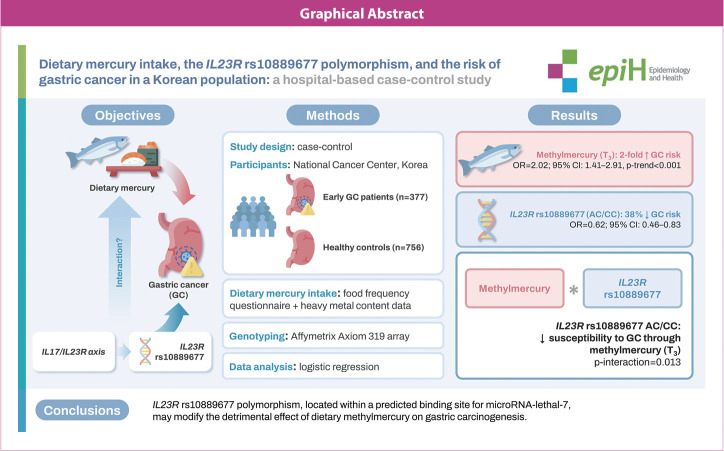


**Table 1.** General characteristics of the study population and their dietary mercury intake, comparing patients with gastric cancer and controls

Characteristics	All (n=1,133)			Men (n=743)			Women (n=390)		
	Controls (n=756)	Cases (n=377)	p-value^1^	Controls (n=497)	Cases (n=246)	p-value^1^	Controls (n=259)	Cases (n=131)	p-value^1^
Age (yr)	53.8±9.0	53.9±9.3	0.947	54.8±8.4	55.0±8.6	0.758	51.9±9.7	51.6±10.1	0.826
Gender									
Men	497 (65.7)	246 (65.3)	0.870	-	-		-	-	
Women	259 (34.3)	131 (34.7)		-	-		-	-	
Body mass index (kg/m^2^)	24.0±2.9	23.9±3.1	0.389	24.5±2.7	24.2±3.0	0.288	23.1±3.1	23.1±3.0	0.984
<23	276 (36.5)	147 (39.0)	0.673	140 (28.2)	84 (34.2)	0.207	136 (52.5)	63 (48.1)	0.727
23-<25	230 (30.4)	107 (28.4)		160 (32.2)	68 (27.6)		70 (27.0)	39 (29.8)	
≥25	249 (32.9)	122 (32.4)		197 (39.6)	94 (38.2)		52 (20.1)	28 (21.4)	
Missing	1 (0.2)	1 (0.2)		0 (0.0)	0 (0.0)		1 (0.4)	1 (0.7)	
*Helicobacter pylori* infection									
Negative	292 (38.6)	28 (7.4)	<0.001	175 (35.2)	16 (6.5)	<0.001	117 (45.2)	12 (9.2)	<0.001
Positive	464 (61.4)	349 (92.6)		322 (64.8)	230 (93.5)		142 (54.8)	119 (90.8)	
Family history of gastric cancer in first-degree relatives									
No	659 (87.2)	299 (79.3)	<0.001	424 (85.3)	190 (77.2)	0.006	235 (90.7)	109 (83.2)	0.030
Yes	95 (12.6)	77 (20.4)		71 (14.3)	55 (22.4)		24 (9.3)	22 (16.8)	
Missing	2 (0.2)	1 (0.3)		2 (0.4)	1 (0.2)		0 (0.0)	0 (0.0)	
Regular exercise									
Yes	424 (56.1)	136 (36.1)	<0.001	279 (56.1)	100 (40.6)	<0.001	145 (56.0)	36 (27.5)	<0.001
No	329 (43.5)	241 (63.9)		215 (43.3)	146 (59.4)		114 (44.0)	95 (72.5)	
Missing	3 (0.4)	0 (0.0)		3 (0.6)	0 (0.0)		0 (0.0)	0 (0.0)	
Smoking status									
Never smokers	344 (45.5)	151 (40.0)	<0.001	96 (19.3)	34 (13.8)	<0.001	248 (95.8)	117 (89.4)	0.038
Ex-smokers	258 (34.1)	110 (29.2)		251 (50.5)	103 (41.9)		7 (2.7)	7 (5.3)	
Current smokers	154 (20.4)	116 (30.8)		150 (30.2)	109 (44.3)		4 (1.5)	7 (5.3)	
Alcohol drinking status									
Never drinkers	212 (28.0)	112 (39.7)	0.333	81 (16.3)	42 (17.1)	0.330	131 (50.6)	70 (53.4)	0.863
Ex-drinkers	58 (7.7)	37 (9.8)		46 (9.3)	31 (12.6)		12 (4.6)	6 (4.6)	
Current drinkers	486 (64.3)	228 (60.5)		370 (74.4)	173 (70.3)		116 (44.8)	55 (42.0)	
Education									
Less than college	334 (44.2)	289 (75.8)	<0.001	188 (37.8)	187 (76.0)	<0.001	146 (56.4)	102 (77.9)	<0.001
College and higher	392 (51.8)	87 (23.1)		281 (56.5)	58 (23.6)		111 (42.9)	29 (22.1)	
Missing	30 (4.0)	1 (0.1)		28 (5.7)	1 (0.4)		2 (0.7)	0 (0.0)	
Monthly income (×10,000 Korean won/mo)									
<200	132 (17.5)	120 (31.8)	<0.001	74 (14.9)	78 (31.7)	<0.001	58 (22.4)	42 (32.1)	0.050
200-<400	313 (41.4)	132 (35.0)		217 (43.7)	94 (38.2)		96 (37.1)	38 (29.0)	
≥400	247 (32.7)	88 (23.3)		153 (30.8)	50 (20.3)		94 (36.3)	38 (29.0)	
Missing	64 (8.4)	37 (9.9)		53 (10.6)	24 (9.8)		11 (4.2)	13 (9.9)	
Total energy intake (kcal/day)	1,717.3±546.9	1,925.2±611.9	<0.001	1,765.5±542.3	2,033.9±635.8	<0.001	1,624.9±544.9	1,721.1±506.4	0.093
Dietary total mercury, crude (µg/day)	13.2±4.2	14.8±4.5	<0.001	13.4±4.1	15.6±4.7	<0.001	12.6±4.4	13.3±3.7	0.110
Dietary total mercury, energy-adjusted (µg/day)^2^	13.7±2.4	14.0±2.3	0.033	13.6±2.0	14.1±2.5	0.011	13.8±3.1	13.9±2.1	0.816
Dietary methylmercury, crude (µg/day)	11.8±4.2	14.1±4.5	<0.001	12.5±4.1	15.0±4.6	<0.001	10.5±4.0	12.4±3.8	<0.001
Dietary methylmercury, energy-adjusted (µg/day)^2^	12.4±3.3	13.5±3.5	<0.001	12.8±3.0	13.7±3.6	<0.001	11.5±3.8	13.1±3.2	<0.001
*IL23R* rs10889677 genotype									
A/A	361 (47.7)	218 (57.8)	0.004	241 (48.5)	139 (56.5)	0.103	120 (46.3)	79 (60.3)	0.019
A/C	331 (43.8)	127 (33.7)		219 (44.1)	89 (36.2)		112 (43.2)	38 (29.0)	
C/C	64 (8.5)	32 (8.5)		37 (7.4)	18 (7.3)		27 (10.4)	14 (10.7)	

Values are presented as number (%) or mean±standard deviation.

1 The comparisons were made between gastric cancer cases and controls using the chi-square test for categorical variables and the Student t-test for continuous variables.

2 Dietary total mercury and methylmercury were adjusted for total energy intake using the residual method.

**Table 2.** Association between dietary mercury intake and the risk of gastric cancer^1^

Dietary mercury (µg/day)	Controls	Cases	Model I	Model II	Model III
Total mercury^2^					
Total (n=1,133)					
T_1_ (<12.78)	252 (33.3)	105 (27.9)	1.00 (reference)	1.00 (reference)	1.00 (reference)
T_2_ (12.78-14.46)	252 (33.3)	126 (33.4)	1.20 (0.88, 1.64)	1.29 (0.91, 1.82)	1.42 (0.99, 2.04)
T_3_ (≥14.46)	252 (33.3)	146 (38.7)	1.39 (1.02, 1.89)	1.38 (0.98, 1.93)	1.38 (0.97, 1.97)
p for trend^3^			0.035	0.068	0.088
Men (n=743)					
T_1_ (<12.90)	166 (33.4)	71 (28.9)	1.00 (reference)	1.00 (reference)	1.00 (reference)
T_2_ (12.90-14.39)	166 (33.4)	74 (30.1)	1.04 (0.71, 1.54)	1.19 (0.77, 1.85)	1.41 (0.89, 2.24)
T_3_ (≥14.39)	165 (33.2)	101 (41.1)	1.43 (0.99, 2.08)	1.55 (1.02, 2.35)	1.73 (1.11, 2.68)
p for trend^3^			0.054	0.040	0.015
Women (n=390)					
T_1_ (<12.51)	87 (33.6)	31 (23.7)	1.00 (reference)	1.00 (reference)	1.00 (reference)
T_2_ (12.51-14.55)	86 (33.2)	54 (41.2)	1.76 (1.03, 3.00)	1.70 (0.94, 3.05)	2.25 (1.04, 4.89)
T_3_ (≥14.55)	86 (33.2)	46 (35.1)	1.50 (0.87, 2.59)	1.39 (0.75, 2.57)	1.96 (0.63, 6.14)
p for trend^3^			0.184	0.345	0.282
Methylmercury^2^					
Total (n=1,133)					
T_1_ (<11.00)	252 (33.3)	81 (21.5)	1.00 (reference)	1.00 (reference)	1.00 (reference)
T_2_ (11.00-13.63)	252 (33.3)	112 (29.7)	1.38 (0.99, 1.93)	1.29 (0.89, 1.86)	1.43 (0.97, 2.11)
T_3_ (≥13.63)	252 (33.3)	184 (48.8)	2.27 (1.66, 3.11)	1.98 (1.40, 2.80)	2.02 (1.41, 2.91)
p for trend^3^			<0.001	<0.001	<0.001
Men (n=743)					
T_1_ (<11.56)	166 (33.4)	62 (25.2)	1.00 (reference)	1.00 (reference)	1.00 (reference)
T_2_ (11.56-13.98)	166 (33.4)	67 (27.2)	1.08 (0.72, 1.62)	1.09 (0.69, 1.70)	1.29 (0.81, 2.08)
T_3_ (≥13.98)	165 (33.2)	117 (47.6)	1.90 (1.30, 2.76)	1.68 (1.11, 2.56)	1.77 (1.14, 2.74)
p for trend^3^			<0.001	0.011	0.010
Women (n=390)					
T_1_ (<9.89)	87 (33.6)	19 (14.5)	1.00 (reference)	1.00 (reference)	1.00 (reference)
T_2_ (9.89-12.97)	86 (33.2)	48 (36.6)	2.56 (1.39, 4.70)	2.26 (1.17, 4.37)	2.58 (1.28, 5.23)
T_3_ (≥12.97)	86 (33.2)	64 (48.9)	3.41 (1.88, 6.16)	2.75 (1.43, 5.30)	2.80 (1.40, 5.62)
p for trend^3^			<0.001	0.004	0.001

Values are presented as number (%) or odds ratio (95% confidence interval).

1 Model I: crude model; Model II: adjusted for age (continuous), gender (unadjusted in the gender-stratified analysis), body mass index (<23, 23-<25, or ≥25 kg/m^2^), smoking status (current-, ex-, or non-smoker), drinking status (current-, ex-, or non-drinker), physical activity (yes or no), education level (less than college or college and higher), income (<200, 200-<400, or ≥400 [×10,000 Korean won/mo]), and first-degree family history of gastric cancer (yes or no); Model III: additionally adjusted for *Helicobacter pylori* infection (positive or negative).

2 The values are presented as tertiles of dietary total mercury or methylmercury intake, which were adjusted for total energy intake using a linear residual regression method.

3 Test for trend was calculated with the median intake for each category of dietary mercury as a continuous variable.

**Table 3.** Association between *IL23R* rs10889677 genetic polymorphism and the risk of gastric cancer^1^

*IL23R* rs10889677	Inheritance model	Controls	Cases	Model I	Model II	Model III
Total (n=1,133)	Dominant model					
	AA	361 (47.8)	218 (57.8)	1.00 (reference)	1.00 (reference)	1.00 (reference)
	AC/CC	395 (52.2)	159 (42.2)	0.67 (0.52, 0.86)	0.68 (0.52, 0.89)	0.62 (0.46, 0.83)
	Allelic model					
	A	1,053 (69.6)	563 (74.7)	1.00 (reference)	1.00 (reference)	1.00 (reference)
	C	459 (30.4)	191 (25.3)	0.78 (0.64, 0.95)	0.80 (0.64, 0.99)	0.74 (0.59, 0.93)
Men (n=743)	Dominant model					
	AA	241 (48.5)	139 (56.5)	1.00 (reference)	1.00 (reference)	1.00 (reference)
	AC/CC	256 (51.5)	107 (43.5)	0.73 (0.53, 0.99)	0.70 (0.50, 0.99)	0.61 (0.42, 0.88)
	Allelic model					
	A	701 (70.5)	367 (74.6)	1.00 (reference)	1.00 (reference)	1.00 (reference)
	C	293 (29.5)	125 (25.4)	0.82 (0.64, 1.04)	0.79 (0.60, 1.04)	0.71 (0.53, 0.95)
Women (n=390)	Dominant model					
	AA	120 (46.3)	79 (60.3)	1.00 (reference)	1.00 (reference)	1.00 (reference)
	AC/CC	139 (53.7)	52 (39.7)	0.57 (0.37, 0.87)	0.60 (0.38, 0.96)	0.60 (0.36, 0.99)
	Allelic model					
	A	352 (68.0)	196 (74.8)	1.00 (reference)	1.00 (reference)	1.00 (reference)
	C	166 (32.0)	66 (25.2)	0.71 (0.51, 1.00)	0.77 (0.54, 1.12)	0.77 (0.52, 1.15)

Values are presented as number (%) or odds ratio (95% confidence interval).

1 Model I: crude model; Model II: adjusted for age (continuous), gender (unadjusted in the gender-stratified analysis), body mass index (<23, 23-<25, or ≥25 kg/m^2^), smoking status (current-, ex-, or non-smoker), drinking status (current-, ex-, or non-drinker), physical activity (yes or no), education level (less than college or college and higher), income (<200, 200-<400, or ≥400 [×10,000 Korean won/mo]), and first-degree family history of gastric cancer (yes or no); Model III: additionally adjusted for *Helicobacter pylori* infection (positive or negative).

**Table 4.** Association between the interaction of *IL23R* rs10889677 polymorphism with dietary total mercury and the risk of gastric cancer^1^

Dominant model	Dietary mercury (µg/day)	Controls	Cases	Model I	Model II	Model III
*IL23R* (rs10889677)						
Total (n=1,133)						
AA	T_1_ (<12.78)	112 (14.8)	55 (14.6)	1.00 (reference)	1.00 (reference)	1.00 (reference)
	T_2_ (12.78-14.46)	127 (16.8)	77 (20.4)	1.24 (0.80, 1.90)	1.47 (0.91, 2.36)	1.60 (0.96, 2.65)
	T_3_ (≥14.46)	122 (16.1)	86 (22.8)	1.44 (0.94, 2.20)	1.40 (0.87, 2.24)	1.36 (0.82, 2.24)
AC/CC	T_1_ (<12.78)	140 (18.5)	50 (13.3)	0.73 (0.46, 1.15)	0.79 (0.48, 1.30)	0.71 (0.42, 1.21)
	T_2_ (12.78-14.46)	125 (16.5)	49 (13.0)	0.80 (0.50, 1.27)	0.83 (0.50, 1.37)	0.81 (0.47, 1.39)
	T_3_ (≥14.46)	130 (17.2)	60 (15.9)	0.94 (0.60, 1.47)	1.03 (0.63, 1.68)	0.94 (0.56, 1.59)
p-interaction				0.748	0.896	0.964
Men (n=743)						
AA	T_1_ (<12.90)	79 (15.9)	40 (16.3)	1.00 (reference)	1.00 (reference)	1.00 (reference)
	T_2_ (12.90-14.39)	81 (16.3)	43 (17.5)	1.05 (0.62, 1.78)	1.25 (0.69, 2.28)	1.44 (0.77, 2.71)
	T_3_ (≥14.39)	81 (16.3)	56 (22.8)	1.37 (0.82, 2.28)	1.31 (0.74, 2.32)	1.32 (0.72, 2.43)
AC/CC	T_1_ (<12.90)	87 (17.5)	31 (12.6)	0.70 (0.40, 1.23)	0.64 (0.35, 1.20)	0.51 (0.27, 0.99)
	T_2_ (12.90-14.39)	85 (17.1)	31 (12.6)	0.72 (0.41, 1.26)	0.72 (0.38, 1.35)	0.68 (0.35, 1.33)
	T_3_ (≥14.39)	84 (16.9)	45 (18.3)	1.06 (0.63, 1.79)	1.18 (0.65, 2.13)	1.17 (0.63, 2.18)
p-interaction				0.790	0.398	0.150
Women (n=390)						
AA	T_1_ (<12.51)	35 (13.5)	17 (13.0)	1.00 (reference)	1.00 (reference)	1.00 (reference)
	T_2_ (12.51-14.55)	43 (16.6)	31 (23.7)	1.48 (0.71, 3.11)	1.61 (0.70, 3.68)	1.84 (0.73, 4.59)
	T_3_ (≥14.55)	42 (16.2)	31 (23.7)	1.52 (0.72, 3.19)	1.59 (0.69, 3.66)	1.58 (0.64, 3.89)
AC/CC	T_1_ (<12.51)	52 (20.1)	14 (10.7)	0.55 (0.24, 1.27)	0.68 (0.27, 1.69)	0.80 (0.30, 2.14)
	T_2_ (12.51-14.55)	43 (16.6)	23 (17.6)	1.10 (0.51, 2.38)	1.19 (0.51, 2.77)	1.35 (0.53, 3.42)
	T_3_ (≥14.55)	44 (17.0)	15 (11.5)	0.70 (0.31, 1.60)	0.71 (0.28, 1.78)	0.61 (0.23, 1.62)
p-interaction				0.780	0.538	0.348

Values are presented as number (%) or odds ratio (95% confidence interval).

1 Model I: crude model; Model II: adjusted for age (continuous), gender (unadjusted in the gender-stratified analysis), body mass index (<23, 23-<25, or ≥25 kg/m^2^), smoking status (current-, ex-, or non-smoker), drinking status (current-, ex-, or non-drinker), physical activity (yes or no), education level (less than college or college and higher), income (<200, 200-<400, or ≥400 [×10,000 Korean won/mo]), and first-degree family history of gastric cancer (yes or no); Model III: additionally adjusted for *Helicobacter pylori* infection (positive or negative).

**Table 5.** Association between the interaction of *IL23R* rs10889677 polymorphism with dietary methylmercury and the risk of gastric cancer^1^

Dominant model	Dietary methylmercury (µg/day)	Controls	Cases	Model I	Model II	Model III
*IL23R* (rs10889677)						
Total (n=1,133)						
AA	T_1_ (<11.00)	126 (16.7)	39 (10.3)	1.00 (reference)	1.00 (reference)	1.00 (reference)
	T_2_ (11.00-13.63)	113 (14.9)	59 (15.7)	1.69 (1.05, 2.72)	1.58 (0.94, 2.67)	1.78 (1.03, 3.09)
	T_3_ (≥13.63)	122 (16.1)	120 (31.8)	3.18 (2.05, 4.93)	2.62 (1.62, 4.23)	2.93 (1.77, 4.87)
AC/CC	T_1_ (<11.00)	126 (16.7)	42 (11.1)	1.08 (0.65, 1.78)	1.05 (0.61, 1.80)	1.04 (0.59, 1.83)
	T_2_ (11.00-13.63)	139 (18.4)	53 (14.1)	1.23 (0.76, 1.99)	1.12 (0.66, 1.88)	1.22 (0.71, 2.11)
	T_3_ (≥13.63)	130 (17.2)	64 (17.0)	1.59 (1.00, 2.54)	1.43 (0.86, 2.39)	1.30 (0.76, 2.21)
p-interaction				0.016	0.047	0.013
Men (n=743)						
AA	T_1_ (<11.56)	85 (17.1)	33 (13.4)	1.00 (reference)	1.00 (reference)	1.00 (reference)
	T_2_ (11.56-13.98)	79 (15.9)	33 (13.4)	1.08 (0.61, 1.91)	1.18 (0.63, 2.21)	1.57 (0.80, 3.07)
	T_3_ (≥13.98)	77 (15.5)	73 (29.7)	2.44 (1.46, 4.08)	2.05 (1.16, 3.64)	2.28 (1.25, 4.17)
AC/CC	T_1_ (<11.56)	81 (16.3)	29 (11.8)	0.92 (0.51, 1.65)	0.90 (0.47, 1.72)	0.86 (0.44, 1.69)
	T_2_ (11.56-13.98)	87 (17.5)	34 (13.8)	1.01 (0.57, 1.77)	0.92 (0.49, 1.72)	0.97 (0.50, 1.87)
	T_3_ (≥13.98)	88 (17.7)	44 (17.9)	1.29 (0.75, 2.21)	1.17 (0.64, 2.15)	1.10 (0.59, 2.07)
p-interaction				0.121	0.337	0.252
Women (n=390)						
AA	T_1_ (<9.89)	40 (15.4)	9 (6.9)	1.00 (reference)	1.00 (reference)	1.00 (reference)
	T_2_ (9.89-12.97)	39 (15.1)	26 (19.8)	2.96 (1.23, 7.12)	2.91 (1.12, 7.58)	2.67 (0.95, 7.50)
	T_3_ (≥12.97)	41 (15.8)	44 (33.6)	4.77 (2.06, 11.04)	3.91 (1.55, 9.91)	4.64 (1.68, 12.81)
AC/CC	T_1_ (<9.89)	47 (18.2)	10 (7.6)	0.95 (0.35, 2.56)	1.04 (0.36, 3.06)	0.98 (0.31, 3.08)
	T_2_ (9.89-12.97)	47 (18.2)	22 (16.8)	2.08 (0.86, 5.03)	1.88 (0.72, 4.91)	2.60 (0.92, 7.36)
	T_3_ (≥12.97)	45 (17.4)	20 (15.3)	1.98 (0.81, 4.83)	1.81 (0.68, 4.81)	1.50 (0.53, 4.26)
p-interaction				0.168	0.228	0.080

Values are presented as number (%) or odds ratio (95% confidence interval).

1 Model I: crude model; Model II: adjusted for age (continuous), gender (unadjusted in the gender-stratified analysis), body mass index (<23, 23-<25, or ≥25 kg/m^2^), smoking status (current-, ex-, or non-smoker), drinking status (current-, ex-, or non-drinker), physical activity (yes or no), education level (less than college or college and higher), income (<200, 200-<400, or ≥400 [×10,000 Korean won/mo]), and first-degree family history of gastric cancer (yes or no); Model III: additionally adjusted for *Helicobacter pylori* infection (positive or negative).

## Supplementary materials

Supplementary Material 1. Association between *IL23R* rs10889677 genetic polymorphism and the risk of gastric cancer in the codominant model

## References

[1] Sung H, Ferlay J, Siegel RL, Laversanne M, Soerjomataram I, Jemal A (2021). Global cancer statistics 2020: GLOBOCAN estimates of incidence and mortality worldwide for 36 cancers in 185 countries. CA Cancer J Clin.

[2] Kang MJ, Won YJ, Lee JJ, Jung KW, Kim HJ, Kong HJ (2022). Cancer statistics in Korea: incidence, mortality, survival, and prevalence in 2019. Cancer Res Treat.

[3] Qing Y, Li Y, Yang J, Li S, Gu K, Bao Y (2022). Risk assessment of mercury through dietary exposure in China. Environ Pollut.

[4] Stohs SJ, Bagchi D (1995). Oxidative mechanisms in the toxicity of metal ions. Free Radic Biol Med.

[5] Walker EV, Girgis S, Yuan Y, Goodman KJ (2021). Community-driven research in the canadian arctic: dietary exposure to methylmercury and gastric health outcomes. Int J Circumpolar Health.

[6] Maqbool F, Niaz K, Hassan FI, Khan F, Abdollahi M (2017). Immunotoxicity of mercury: pathological and toxicological effects. J Environ Sci Health C Environ Carcinog Ecotoxicol Rev.

[7] Peralta-Videa JR, Lopez ML, Narayan M, Saupe G, Gardea-Torresdey J (2009). The biochemistry of environmental heavy metal uptake by plants: implications for the food chain. Int J Biochem Cell Biol.

[8] Rice KM, Walker EM, Wu M, Gillette C, Blough ER (2014). Environmental mercury and its toxic effects. J Prev Med Public Health.

[9] Fowler J, Tsui MT, Chavez J, Khan S, Ahmed H, Smith L (2021). Methyl mercury triggers endothelial leukocyte adhesion and increases expression of cell adhesion molecules and chemokines. Exp Biol Med (Maywood).

[10] Xue Z, Xue H, Jiang J, Lin B, Zeng S, Huang X (2014). Chronic atrophic gastritis in association with hair mercury level. Tumour Biol.

[11] Kim H, Lee J, Woo HD, Kim DW, Oh JH, Chang HJ (2020). Dietary mercury intake and colorectal cancer risk: a case-control study. Clin Nutr.

[12] Hong YS, Kim YM, Lee KE (2012). Methylmercury exposure and health effects. J Prev Med Public Health.

[13] Skalny AV, Aschner M, Sekacheva MI, Santamaria A, Barbosa F, Ferrer B (2022). Mercury and cancer: where are we now after two decades of research?. Food Chem Toxicol.

[14] Yuan W, Yang N, Li X (2016). Advances in understanding how heavy metal pollution triggers gastric cancer. Biomed Res Int.

[15] Khan D, Ansar Ahmed S (2015). Regulation of IL-17 in autoimmune diseases by transcriptional factors and microRNAs. Front Genet.

[16] Stockinger B, Hirota K, Duarte J, Veldhoen M (2011). External influences on the immune system via activation of the aryl hydrocarbon receptor. Semin Immunol.

[17] Gutiérrez-Vázquez C, Quintana FJ (2018). Regulation of the immune response by the aryl hydrocarbon receptor. Immunity.

[18] Hemdan NY, Abu El-Saad AM, Sack U (2013). The role of T helper (TH)17 cells as a double-edged sword in the interplay of infection and autoimmunity with a focus on xenobiotic-induced immunomodulation. Clin Dev Immunol.

[19] Weinhouse C, Perez L, Ryde I, Goodrich JM, Miranda JJ, Hsu-Kim H (2022). Epigenetic biomarkers of autoimmune risk and protective antioxidant signaling in methylmercury-exposed adults. bioRxiv [Preprint].

[20] Chen B, Zeng Z, Xu L, Wu X, Yu J, Xue L (2011). IL23R +2199A/C polymorphism is associated with decreased risk of certain subtypes of gastric cancer in Chinese: a case-control study. Cancer Epidemiol.

[21] El-Gedamy M, Şentürk M (2023). Chemokines updates.

[22] Yao J, Liu L, Yang M (2014). Interleukin-23 receptor genetic variants contribute to susceptibility of multiple cancers. Gene.

[23] Mosallaei M, Simonian M, Esmaeilzadeh E, Bagheri H, Miraghajani M, Salehi AR (2019). Single nucleotide polymorphism rs10889677 in miRNAs Let-7e and Let-7f binding site of IL23R gene is a strong colorectal cancer determinant: report and meta-analysis. Cancer Genet.

[24] Zhou S, Ruan Y, Yu H, Chen Y, Yao Y, Ma Y (2013). Functional IL23R rs10889677 genetic polymorphism and risk of multiple solid tumors: a meta-analysis. PLoS One.

[25] Dong K, Xu Y, Yang Q, Shi J, Jiang J, Chen Y (2017). Associations of functional microRNA binding site polymorphisms in IL23/Th17 inflammatory pathway genes with gastric cancer risk. Mediators Inflamm.

[26] Jia ZF, Cao DH, Wu YH, Jin MS, Pan YC, Cao XY (2019). Lethal-7-related polymorphisms are associated with susceptibility to and prognosis of gastric cancer. World J Gastroenterol.

[27] He B, Pan B, Pan Y, Wang X, Zhou L, Sun H (2019). Polymorphisms of IL-23R predict survival of gastric cancer patients in a Chinese population. Cytokine.

[28] Omrane I, Baroudi O, Bougatef K, Mezlini A, Abidi A, Medimegh I (2014). Significant association between IL23R and IL17F polymorphisms and clinical features of colorectal cancer. Immunol Lett.

[29] Li M, Yue C, Jin G, Guo H, Ma H, Wang G (2019). Rs1884444 variant in IL23R gene is associated with a decreased risk in esophageal cancer in Chinese population. Mol Carcinog.

[30] Kim JH, Lee J, Choi IJ, Kim YI, Kim J (2021). Dietary patterns and gastric cancer risk in a Korean population: a case-control study. Eur J Nutr.

[31] Ahn Y, Kwon E, Shim JE, Park MK, Joo Y, Kimm K (2007). Validation and reproducibility of food frequency questionnaire for Korean genome epidemiologic study. Eur J Clin Nutr.

[32] Kim DW, Woo HD, Joo J, Park KS, Oh SY, Kwon HJ (2014). Estimated long-term dietary exposure to lead, cadmium, and mercury in young Korean children. Eur J Clin Nutr.

[33] Jo S, Woo HD, Kwon HJ, Oh SY, Park JD, Hong YS (2015). Estimation of the biological half-life of methylmercury using a population toxicokinetic model. Int J Environ Res Public Health.

[34] Kim W, Woo HD, Lee J, Choi IJ, Kim YW, Sung J (2016). Dietary folate, one-carbon metabolism-related genes, and gastric cancer risk in Korea. Mol Nutr Food Res.

[35] Park B, Yang S, Lee J, Woo HD, Choi IJ, Kim YW (2019). Genomewide association of genetic variation in the PSCA gene with gastric cancer susceptibility in a Korean population. Cancer Res Treat.

[36] Willett WC, Howe GR, Kushi LH (1997). Adjustment for total energy intake in epidemiologic studies. Am J Clin Nutr.

[37] Dewayani A, Fauzia KA, Alfaray RI, Waskito LA, Doohan D, Rezkitha YA (2021). The roles of IL-17, IL-21, and IL-23 in the Helicobacter pylori infection and gastrointestinal inflammation: a review. Toxins (Basel).

[38] Caruso R, Pallone F, Monteleone G (2007). Emerging role of IL-23/IL-17 axis in H pylori-associated pathology. World J Gastroenterol.

[39] Zefferino R, Piccoli C, Ricciardi N, Scrima R, Capitanio N (2017). Possible mechanisms of mercury toxicity and cancer promotion: involvement of gap junction intercellular communications and inflammatory cytokines. Oxid Med Cell Longev.

[40] Ni B, Chen S, Xie H, Ma H (2014). Functional polymorphisms in interleukin-23 receptor and susceptibility to esophageal squamous cell carcinoma in Chinese population. PLoS One.

[41] Wang L, Liu W, Jiang W, Lin J, Jiang Y, Li B (2012). A miRNA binding site single-nucleotide polymorphism in the 3´-UTR region of the IL23R gene is associated with breast cancer. PLoS One.

[42] Chen Z, Laurence A, O’Shea JJ (2007). Signal transduction pathways and transcriptional regulation in the control of Th17 differentiation. Semin Immunol.

[43] Volpe E, Servant N, Zollinger R, Bogiatzi SI, Hupé P, Barillot E (2008). A critical function for transforming growth factor-beta, interleukin 23 and proinflammatory cytokines in driving and modulating human T(H)-17 responses. Nat Immunol.

[44] Iwakura Y, Ishigame H (2006). The IL-23/IL-17 axis in inflammation. J Clin Invest.

[45] Zheng J, Jiang L, Zhang L, Yang L, Deng J, You Y (2012). Functional genetic variations in the IL-23 receptor gene are associated with risk of breast, lung and nasopharyngeal cancer in Chinese populations. Carcinogenesis.

[46] Li Q, Li Q, Chen J, Liu Y, Zhao X, Tan B (2013). Prevalence of Th17 and Treg cells in gastric cancer patients and its correlation with clinical parameters. Oncol Rep.

[47] Voo KS, Wang YH, Santori FR, Boggiano C, Wang YH, Arima K (2009). Identification of IL-17-producing FOXP3+ regulatory T cells in humans. Proc Natl Acad Sci U S A.

[48] You CH, Kim BG, Kim YM, Lee SA, Kim RB, Seo JW (2014). Relationship between dietary mercury intake and blood mercury level in Korea. J Korean Med Sci.

[49] Wang L, Miao C, He Y, Li H, Zhang S, Li K (2022). The influence of heavy metals on gastric tumorigenesis. J Oncol.

[50] Andreoli V, Sprovieri F (2017). Genetic aspects of susceptibility to mercury toxicity: an overview. Int J Environ Res Public Health.

