# Supplementary material for: Dietary mercury intake, the IL23R rs10889677 polymorphism, and the risk of gastric cancer in a Korean population: a hospital-based case-control study
Source: Epidemiol Health. 2024 May 21;46:e2024051. doi: 10.4178/epih.e2024051 (PMC11573488; doi:10.4178/epih.e2024051)
Supplement: Supplementary Material 1. — Association between IL23R rs10889677 genetic polymorphism and the risk of gastric cancer in the codominant model [file epih-46-e2024051-Supplementary-1.docx]

**Supplementary Material 1.** Association between *IL23R* rs10889677 genetic polymorphism and the risk of gastric cancer in the codominant model

|  | Inheritance model | Number (%) | | OR (95% CI) | | |
| --- | --- | --- | --- | --- | --- | --- |
|  |  | Controls | Cases | Model I | Model II | Model III |
| *IL23R* rs10889677 |  |  |  |  |  |  |
| Total (n=1,133) | Codominant model |  |  |  |  |  |
|  | AA | 361 (47.7) | 218 (57.8) | 1.00 (reference) | 1.00 (reference) | 1.00 (reference) |
|  | AC | 331 (43.8) | 127 (33.7) | 0.64 (0.49–0.83) | 0.64 (0.48–0.85) | 0.59 (0.43–0.80) |
|  | CC | 64 (8.5) | 32 (8.5) | 0.83 (0.53–1.31) | 0.89 (0.54–1.48) | 0.79 (0.46–1.36) |
| Men (n=743) | Codominant model |  |  |  |  |  |
|  | AA | 241 (48.5) | 139 (56.5) | 1.00 (reference) | 1.00 (reference) | 1.00 (reference) |
|  | AC | 219 (44.1) | 89 (36.2) | 0.71 (0.51–0.97) | 0.69 (0.48–0.98) | 0.61 (0.41–0.89) |
|  | CC | 37 (7.4) | 18 (7.3) | 0.84 (0.46–1.54) | 0.79 (0.40–1.56) | 0.64 (0.31–1.30) |
| Women (n=390) | Codominant model |  |  |  |  |  |
|  | AA | 120 (46.3) | 79 (60.3) | 1.00 (reference) | 1.00 (reference) | 1.00 (reference) |
|  | AC | 112 (43.2) | 38 (29.0) | 0.52 (0.32–0.82) | 0.53(0.32–0.88) | 0.53(0.31–0.91) |
|  | CC | 27 (10.4) | 14 (10.7) | 0.79 (0.39–1.59) | 0.96 (0.43–2.13) | 0.97 (0.41–2.34) |

OR, odds ratio; CI, confidence interval; Model I, crude model; Model II, adjusted for age (continuous), gender (unadjusted in the gender-stratified analysis), body mass index (<23, 23–<25, or ≥25 kg/m^2^), smoking status (current-, ex-, or non-smoker), drinking status (current-, ex-, or non-drinker), physical activity (yes or no), education level (less than college or college and higher), income (<200, 200–<400, or ≥400 [×10,000 Korean won/mo.]), and first-degree family history of gastric cancer (yes or no); Model III, additionally adjusted for *Helicobacter pylori* infection (positive or negative).
